# Fascial Ultrasound-Guided Injection: Where Do We Really Inject?

**DOI:** 10.7759/cureus.78867

**Published:** 2025-02-11

**Authors:** Kousuke Shiwaku, Carmelo Pirri, Hidenori Otsubo, Tomoaki Kamiya, Andrea Porzionato, Gakuto Nakao, Atsushi Teramoto, Carla Stecco

**Affiliations:** 1 Orthopaedic Surgery, Sapporo Medical University, Sapporo, JPN; 2 Neurosciences, Padova University, Padova, ITA; 3 Orthopaedic Surgery, Sapporo Sports Clinic, Sapporo, JPN; 4 Anatomy, Sapporo Medical University, Sapporo, JPN; 5 Graduate School of Health Sciences, Sapporo Medical University, Sapporo, JPN

**Keywords:** aponeurotic fascia, epimysium, hydrodissection, hydrorelease, myofascial pain syndrome, superficial fascia, ultrasound

## Abstract

Background: The relationship between human anatomy on ultrasonography (US) and dissection is unclear.Therefore, we investigated the precise location of a solution injected into eight legs from five fresh-frozen specimens between the fascial layers of two aponeurotic fascial (APF) regions using US.

Methods: The US-guided injection target points were the fascial lata-distal, crural fascia-proximal, and crural fascia-distal. The operator searched the optimal visualization area of two close fascial layers of the two APF regions; 2.5 mL of 0.9% saline was injected through US guidance. The layers with the solution were categorized as above the APF (between the superficial fascia and APF), intra-APF, between the APF and epimysium (EPI), or intra-muscle (under the EPI).

Results: A small amount of solution was identified within the intra-APF region, whereas a substantial amount was observed above the APF, between the APF and EPI, and intramuscularly. Regarding the fascia lata-distal and crural fascia-distal, a substantial volume of solution was observed between the APF and EPI in all cases. For the crural fascia-proximal, the solution was observed above the APF in 25% of cases and intramuscularly in 75% of cases.

Conclusions: Solution distribution may be associated with whether the muscle fibers are directly inserted into the APF and the absence of EPI in each area.

## Introduction

Hydrorelease (HR) or hydrodissection is the ultrasound (US)-guided injection of a solution into the fascia without anesthesia [1]. It is indicated for conditions, such as nerve disorders and myofascial pain syndrome (MPS) [1]. For HR for MPS, the solution is injected into the inter-fascia, intra-aponeurotic fascia (APF), or intra-muscle [2] to restore normal gliding between layers [3].

Several reports discuss the clinical outcomes related to significant pain reduction after fascial injection for MPS and fascia-related disorders [2,4-6]. A study revealed a significant improvement in the shoulder range of motion (ROM) of flexion, extension, abduction, external rotation, and internal rotation after HR of the coracohumeral ligament in patients with a global limitation of shoulder ROM [6]. Some histological studies have revealed that the APF comprises two, three, or four layers [7]. Notably, a few studies have aimed to ascertain the solution distribution following US-guided inter-myofascial or intra-articular injection [8,9]. However, no cadaveric study has examined the precise distribution of the solution following injection between the layers of the two APFs through US. Therefore, no clear evidence exists regarding the relationship between the human anatomy as seen on US and dissection. Thus, in this cadaveric study, we aimed to investigate the exact location of the solution injected between the fascial layers of two APFs through US. The information is useful when we perform a fascial injection or speculate fascial pathology in clinical settings.

## Materials and methods

Specimen preparation

This study was conducted in accordance with the Declaration of Helsinki. The study protocol to retrieve, use, and dispose of fresh-frozen human cadaveric legs was approved by the Ethics Committee of Padova University. This study used eight legs from five fresh-frozen specimens (mean age at death, 82.0 years; range, 71-90 years), with no evident scars from previous surgery or leg trauma, donated to the university anatomy program. Appropriate informed consent was obtained for cadavers donated to the Institute of Human Anatomy of the University of Padova according to the “Body Donation Programme” of the Institute of Anatomy of the University of Padova [10]. This study was conducted in accordance with the principles of ethical guidelines and laws that pertain to the use of human cadaveric donors in anatomical research. The specimens were thawed at room temperature for at least 24 h before testing. Each specimen was subsequently placed in the supine position and maintained in a wet state to preserve the tissue integrity during the test.

US-guided injection

A 6-15-MHz linear transducer (Edge II, Sonosite, FUJIFILM, WA, USA) was used for US imaging. A screen resolution of 1680 × 1050 pixels and a sound speed (c) of 1540 m/s were used in this study. An orthopedic specialist with US expertise performed US examinations, while another specialist, also trained in US examinations, observed simultaneously. 

Target Points

**Figure 1:**
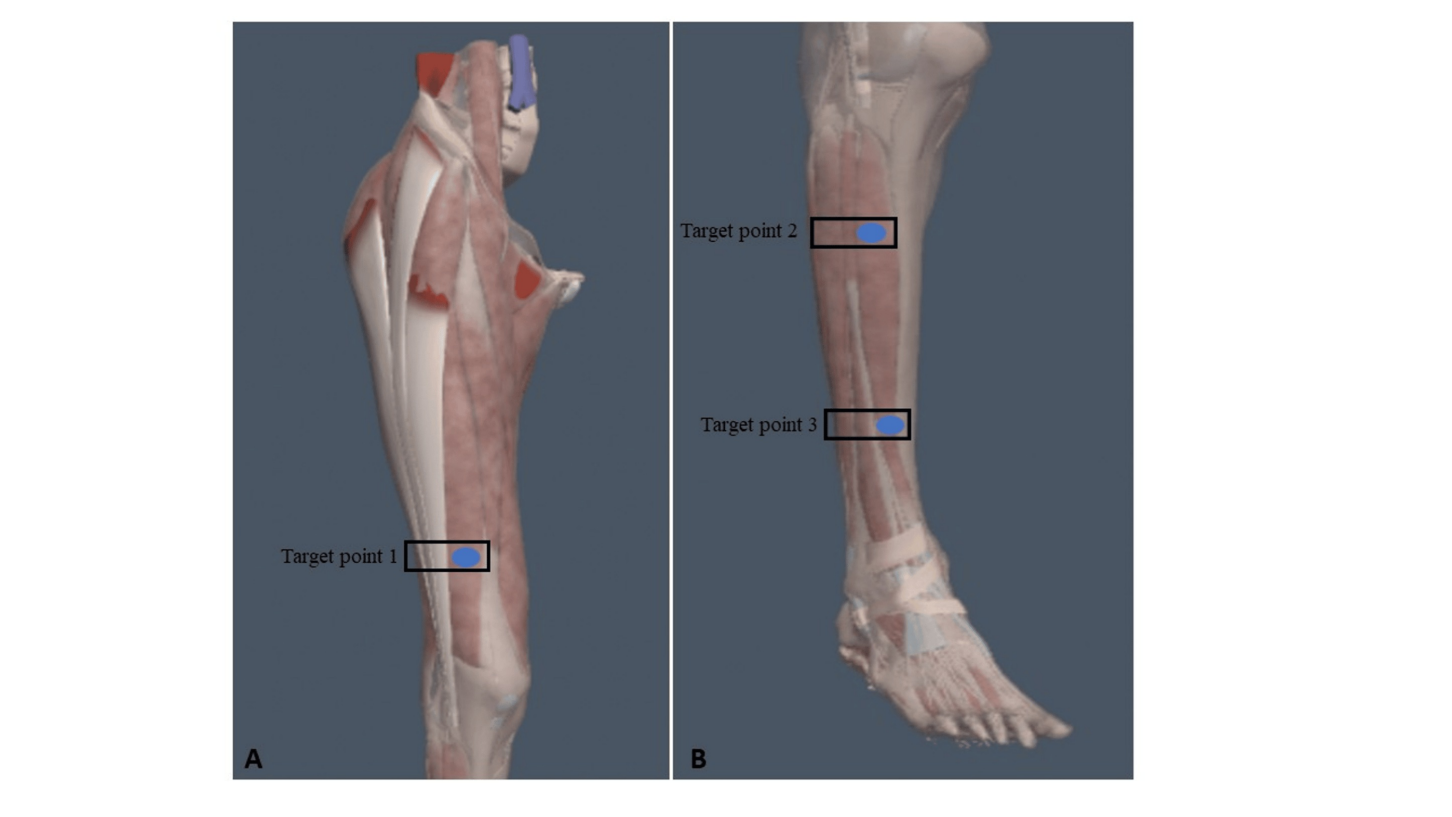
Target points of the ultrasound-guided injection

The target points were determined following the protocols proposed by Pirri et al. [7,11]:

Anterior (Ant) 2 of the fascia lata (Target point 1) : The anterior region is at level 2. The probe was moved downwards from the anterior superior iliac spine to the rectus femoris and vastus intermedius muscles until the sartorius was no longer visible. The vastus medialis and lateralis appeared medially and laterally, respectively (Figure 1).

Ant 1 of the crural fascia (Target point 2): The anterior region is at level 1. The probe was moved downwards, located beneath the crest of the tibia in the anterior-proximal third of the leg (Figure 1).

Ant 2 of the crural fascia (Target point 3):The anterior region is at level 2. The probe was moved downwards from Ant 1 of the crural fascia, tracing the path along the anterior tibialis muscle at the level of the anterior middle third of the leg. It was then shifted laterally until the belly of the anterior tibialis muscle was centrally positioned on the screen (Figure 1). The fascia lata distal is the area around the distal one-third of the anterolateral section of the fascia lata. The crural fascia-proximal is the area around the proximal third of the anterior section of the crural fascia. The crural fascia-distal is the area around the distal third of the anterior section of the crural fascia.

Injection Procedure

**Figure 2:**
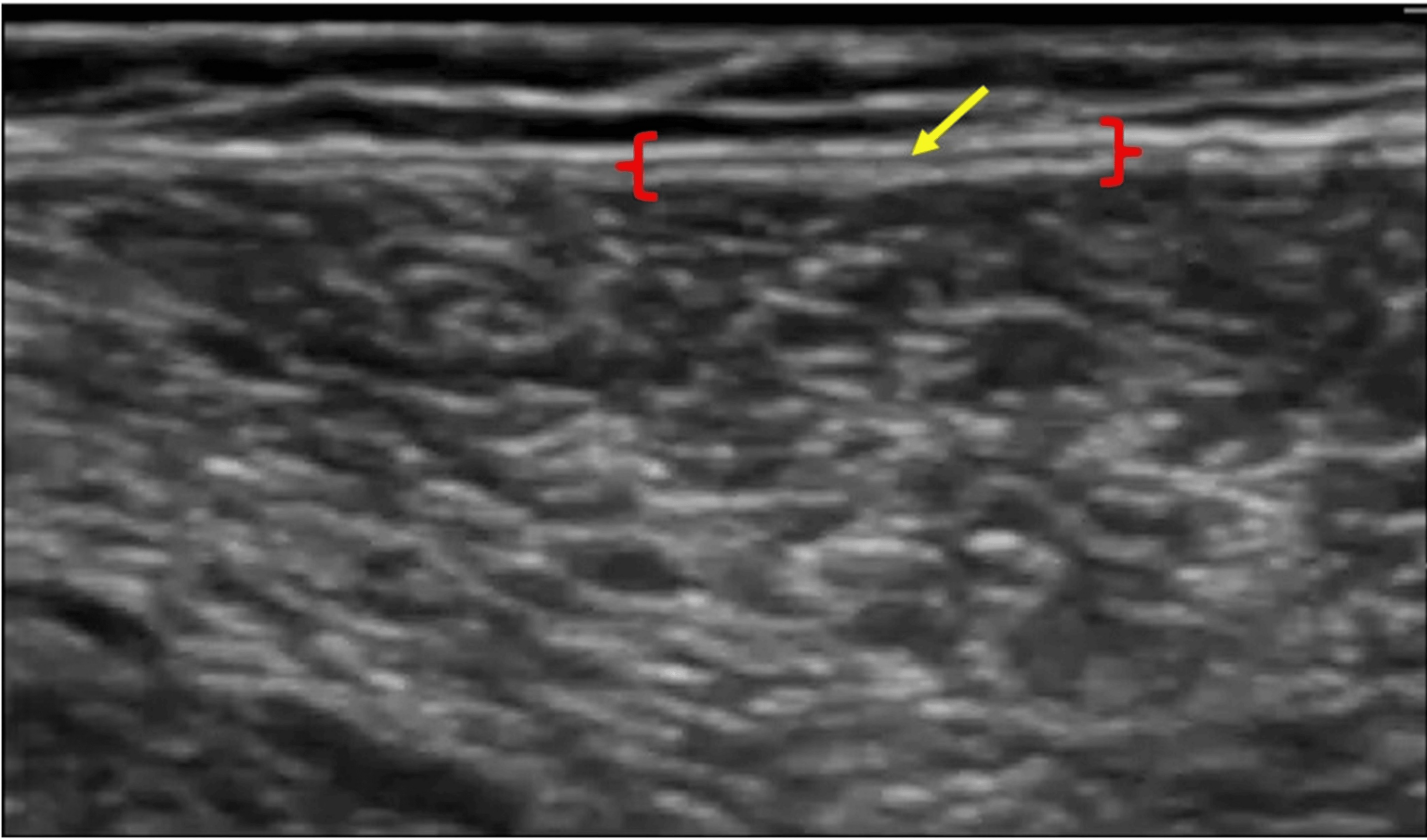
Two close fascial layers in the distal part of the fascia lata Two close fascial layers in the distal part of the fascia lata Arrow: the tip of the needle for injection. {}: two close fascial layers. Note: this is a short-axis ultrasound image.

The operator searched the area where two close fascial layers of the two APFs could be optimally visualized using US [12] after searching widely for the continuation of the APF under skin and fatty areas and where it seemed possible to inject into the layer between the two close fascial layers. Moreover, a 2.5-mL injection of 0.9% saline solution mixed with black or blue ink was guided through US imaging using a 40-mm 23G needle. During the injection, the needle tip was kept between the two fascial layers following the in-plane technique (Figure 2).

Confirmation by dissection

**Figure 3:**
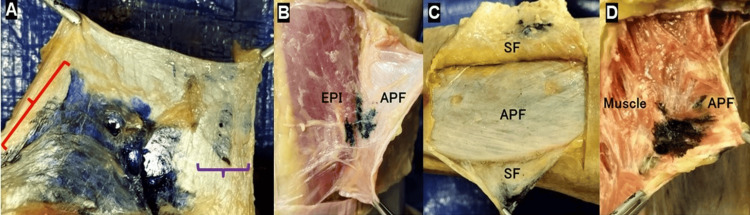
Dissection photographs after an injection with ink (A) Intra-APF (only a small amount, accompanied by a large amount of solution between the APF and EPI); fascia lata-distal. (B) Over the APF (between the SF and APF); crural fascia-proximal. (C) Between the APF and EPI; fascia lata-distal. (d) Intramuscular; crural fascia-proximal. Purple curly bracket ({): only a small amount of solution in the intra-APF region. Red curly bracket ({): a large amount of solution between the APF and EPI.  APF, aponeurotic fascia; EPI, epimysium; SF, superficial fascia.

Dissection was promptly initiated within 1 min of injection and completed in approximately 10 min. The insertion point through the skin and the injection points between the two layers were confirmed. Furthermore, a U-shaped skin incision was made 5 cm from the injection site. Subsequently, the superficial fascia (SF), APF, and epimysium (EPI), if visible, were carefully exposed (Figure 3) until each layer was separated. Then, three observers confirmed the location of the injected solution. The layers where the solution was present were categorized as over-APF (between the SF and APF), intra-APF, APF and EPI, or intra-muscle (under the EPI).

## Results

Only a small amount of solution (~0.833ml) was identified within the intra-APF region; 50.0% was identified in the fascia lata-distal region, and 12.5% in the crural fascia-proximal region (Figure 3A). This was always accompanied by a substantial concentration of the solution located over the APF (between the SF and APF), between the APF and EPI, or intra-muscularly (under EPI). The incidence rates of the small solution volume in the intra-APF space were 50% in the fascia lata-distal, 12.5% in the crural fascia-proximal, and 0% in the crural fascia-distal (Table 1).

**Table 1:** Major Amounts of the Solution in Different Regions Major amounts of the solution in different regions

Site	Cases of major amounts of the solution in different regions	
Fascia lata-distal	Over the APF	0 (0.0%)
	Between the APF and EPI	8 (100.0%)
	Intramuscular	0 (0.0%)
Crural fascia-proximal	Over the APF	2 (25.0%)
	Between the APF and EPI	0 (0.0%)
	Intramuscular	6 (75.0%)
Crural fascia-distal	Over APF	0 (0.0%)
	Between APF and EPI	8 (100.0%)
	Intra muscle	0 (0.0%)

A major amount of the solution was observed over the APF (between the SF and APF), between the APF and EPI, or intramuscularly (Figure 3B-3D). Regarding the fascia lata-distal and crural fascia-distal, a substantial volume of the solution was identified between the APF and EPI in all cases. However, regarding the crural fascia-proximal, the main volume of the solution was observed over the APF (between the SF and APF) in 25% of cases and intramuscularly in 75% of cases (Table 1).

## Discussion

The results indicate that after injection between the two fascial layers, which the operators regarded as the two layers of the APF on US imaging, a small amount of solution was observed in the intra-APF region in only a few cases. Moreover, a larger amount of solution was found in the layer between the APF and EPI at the fascia lata distal and crural fascia-distal and over the APF or intramuscularly at the crural fascia-proximal.

In some cases, the solution was present in the intra-APF region; however, only a small volume of solution was present in this region, and the main amount was present in other layers in all cases. Given the limited space within the multiple layers of the APF, only a small amount of solution can be injected in this region, and most of the solution disperses elsewhere. The efficacy of HR in the layers between the APF and EPI, as well as intramuscularly, has not been thoroughly investigated. The only reported instance involves alterations in post-HR gliding, specifically in the case of HR around the median nerve in the carpal tunnel [3]. Therefore, future research should explore the biomechanical effects of HR to gain a deeper understanding of its impact on gliding.

The variations observed in the solution distribution among the three areas appear to be influenced by whether muscle fibers are directly integrated into the APF and the absence of EPI in each respective area. Therefore, understanding the anatomy of each area can help predict where the solution will end up before the injection. However, in cases where the anatomy is unknown, the solution may disperse over the APF or intramuscularly. Thus, confirming the presence of EPI through the dissection of each area is crucial. Kimura et al. reported that injection aimed at the layer between the two EPIs of the trapezium and rhomboid muscle resulted in the solution being dispersed between the two EPIs [8]. No other studies have investigated the relationship between anatomy and dissection with interfascial injection through US. Further similar investigations are necessary to understand the relationship between the anatomy and dissection of each area.

This cadaveric study demonstrates the challenges of injecting between the two layers of the APF. The results may not perfectly apply and correlate to living humans; therefore, it may be difficult to ascertain the location of the injection. Thus, we suggest that the solution might go to different layers based on the similarity of the US images selected by the operators, depending on where the injection is given. Therefore, more attention should be paid to where the injection is given. To achieve this, a thorough understanding of the relationship between anatomy and dissection on US is essential, especially in each anatomical region. Future clinical and other fundamental studies based on a comprehensive understanding of this relationship may help clarify currently unknown or misunderstood pathologies. We believe these lead to the development of clinical diagnosis and treatment of fascial problems including MPS.

This study has some limitations. First, we used 23G needles because this thickness is commonly used in clinical practice. However, using a thinner needle may have altered the results. Second, we used fresh-frozen cadavers, which do not exhibit in vivo conditions and may contain certain artifacts [13]. Third, while it is common for cadaveric research, a sample size of eight legs may be insufficient for these experiments. Fourth, there may be an observer bias to assess the distribution of the solution. Fifth, because the experiments were performed using specimens from older donors, there may be cases of poor fascial quality and degenerative changes. Although no obvious degenerative change was observed for the specimens of the present study, this may have been a source of bias. Finally, the specimens were sourced from normal cadavers without pathology, indicating that the thickness of the fascia and relationships between layers are representative of the normal human anatomy. However, this may differ from clinical scenarios. 

## Conclusions

Following injection aimed at the layer between the two fascial layers, US examination revealed that the solution may be present in the layer over the APF, in the intra-APF region, between the APF and EPI, or within the muscle. Moreover, the distribution of the solution may be correlated with whether the muscle fibers are directly inserted into the APF and the absence of EPI in each area. Therefore, attention should be paid to the precise injection site.
